# Variation in Pubic Symphysis Fusion Across Primates: Implications for Obstetric Adaptation

**DOI:** 10.1002/ajpa.25064

**Published:** 2025-02-05

**Authors:** Nicole Torres‐Tamayo, Laura T. Buck, Eishi Hirasaki, Todd C. Rae, Lia Betti

**Affiliations:** ^1^ Institute of Evolutionary Medicine University of Zurich Zurich Switzerland; ^2^ Department of Anthropology University College London London UK; ^3^ Research Centre for Evolutionary Anthropology and Palaeoecology, School of Biological and Environmental Sciences Liverpool John Moores University Liverpool UK; ^4^ Center for the Evolutionary Origins of Human Behavior Kyoto University Inuyama Japan; ^5^ School of Life Sciences University of Sussex Brighton UK

**Keywords:** obstetric, obstetrical dilemma, pelvis, primate, pubic symphysis

## Abstract

**Objectives:**

The unfused human pubic symphysis has been interpreted as an obstetric adaptation to facilitate the passage of a large‐brained baby through a relatively small, bipedally adapted pelvis. The degree of fusion of the adult pubic symphysis was evaluated across primate species to gauge whether an open symphysis can be interpreted as an obstetric adaptation in humans and other primates.

**Materials and Methods:**

Symphyseal fusion was assessed in 718 individuals from 67 nonhuman primate species. Variation in fusion in specimens of known ages and sex from four species ( *Galago moholi* , *Macaca mulatta* , *Microcebus murinus*, and *Pan troglodytes* ) was further examined, with detailed analyses of pubic changes by age and sex carried out through logistic regressions in macaques.

**Results:**

Pubic fusion occurs in most primate species. It is observed earlier in life in males than in females in *Ma. mulatta* and *Pa. troglodytes*, only in males in *Mi. murinus*, and does not occur in *Ga. moholi*.

**Discussion:**

While delayed or absent pubic fusion is more prevalent in female primates, suggesting obstetric adaptation, there is no clear relation with childbirth constraints, as fusion is also observed in species experiencing a tight cephalopelvic fit. Other mechanisms might have evolved to facilitate birth in some species, or nonobstetric selective pressures might be counteracting the obstetric advantages of a flexible symphysis. The preservation of an open symphysis throughout life in humans and some other primates, however, can be best interpreted as convergent evolution due to obstetric selection.

## Graphical abstract

Variation in the fusion of the pubic symphysis in primate pelves.

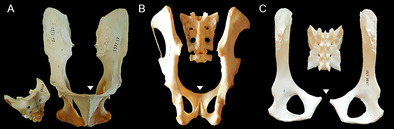

**Summary**

- Many adult primates exhibit pubic symphyseal fusion.
- Fusion is absent or delayed in females, relative to males, in some primate species.
- Sex differences suggest unfused symphyses are an obstetric adaptation that is not unique to humans.

## 1 Introduction

The adult mammalian pelvic girdle is a rigid bony ring, with the two hip bones (ossa coxae) tightly articulated to the sacrum dorsally and most often fused ventrally where the pubic bones meet (Todd 1921a). In humans, however, the pubic bones do not fuse and are instead connected by a fibrocartilaginous joint called the pubic symphysis, with strong ligaments and muscular fibers providing stability by limiting pubic joint movement during bipedal locomotion (Hagen 1974). Hormonal changes during pregnancy, on the other hand, lead to relaxation of tendons and ligaments and softening of joint cartilage, allowing the birth canal to expand during childbirth to facilitate the passage of the fetus (Hagen 1974; MacLennan 1991).

This versatility of the unfused human pubic joint, providing both stability and flexibility, has often been interpreted as an adaptation to alleviate an evolutionary “obstetrical dilemma” (*sensu* Washburn 1960), the conflict between the requirements for bipedalism, favoring a compact pelvis, and for reproduction, which would favor a wide birth canal for particularly large‐brained neonates (e.g., Grunstra et al. 2019; Haeusler et al. 2021; Lovejoy et al. 1997; Lovejoy 2005). The obstetrical dilemma hypothesis holds that the solution to these contrasting requirements has been the development of a larger, rounder birth canal in women compared to men (i.e., sexual dimorphism in the adult pelvis), coupled with a lower proportion of brain growth in utero to ensure the passage of the fetal head through the canal, resulting in secondary altriciality (Washburn 1960). The unfused human pubic joint further serves to increase the flexibility (and, thus, the size) of the pelvic canal during birth, facilitating the passage of the fetus for a successful delivery. This interpretation has meant that humans have been the focus of most investigations of pubic form, relative to that of other primates, but variation across the order is present and may provide insight into understanding the evolution of our obstetrical dilemma.

The adult mammalian pubic joint can take three different forms: (1) the two ossa coxae fuse together in adulthood (synostosis); (2) they remain separate throughout life but articulate tightly via a symphysis; (3) the two pelvic halves do not meet at the symphysis, being connected simply by a ligamentous band or widely separated with no connection at all (Todd 1921a). All these forms have been observed previously in the order Primates. Symphyseal fusion can occur in primates via two different processes: maturational fusion and senescent fusion (Lovejoy et al. 1997). The first process is part of development into adulthood, whereby the fusion of the two pubic bones is the normal endpoint of growth; in this process, the epiphyses of the two pubic bones fuse together into a “median bar” instead of joining to their respective pubic bodies, and only later a complete fusion of the bar and the two pubic bones occurs (Lovejoy et al. 1997). The second process, on the other hand, is an effect of senescence; the pubic epiphyses fuse to their respective pubic bones, leaving a pubic gap into adulthood. In this case, the fusion of the two pubic bones happens later in life, through the type of bone proliferation usually associated with old age and degenerative changes and not as part of skeletal maturation (Lovejoy et al. 1997). Finally, some species of primates appear to have evolved specific mechanisms that prevent the midline fusion of the pubic joint altogether and maintain an open pubic gap even in old age (Lovejoy et al. 1997). Humans fall within this latter group, but it is unclear what other species might have evolved a similar strategy (Lovejoy 2005).

To date, no primate species has been reported to display pubic fusion across all individuals. In those primate species for which pubic fusion has been observed ( *Presbytis rubicunda* , *Macaca mulatta* , *Hylobates lar* , *Pan troglodytes* ), it occurs more frequently in males than in females, potentially forming part of sexually dimorphic pelvic morphology in these species (Rawlins 1975; Tague 1993, 2016) (Table 1).

**TABLE 1:** Proportion of female and male individuals with a fused pubic symphysis in different primate species. Sample size in brackets. Data from: Casteleyn et al. (2012), Lovejoy et al. (1997), Tague (2016).

Species	Fusion in F	Fusion in M
*Aotus azarae*	0% (15)	0% (19)
*Callithrix jacchus*	0% (7)	0% (3)
*Gorilla gorilla*	0% (12)	0% (37)
*Hylobates lar*	27% (37)	67% (32)
*Macaca mulatta*	1% (136)	23% (181)
*Pan troglodytes*	26% (27)	42% (12)
*Presbytis rubicunda*	44% (16)	83% (18)
*Saguinus geoffroyi*	0% (28)	0% (30)
*Saguinus oedipus*	0% (28)	0% (39)
*Trachypithecus cristatus*	0% (21)	0% (15)

In addition to humans, other primate taxa are reported to exhibit no fusion of the pubic bones in adults, including some platyrrhines (*Saguinus oedipus, Callithrix jacchus, Aotus azarae, Sag. geoffroyi*) and the cercopithecoid *Trachypithecus cristatus* ; there are conflicting reports for the hominoid *Gorilla gorilla* (Lovejoy et al. 1997; Todd 1921a).

Explanations of the variation in pubic fusion in primates have varied widely. For example, locomotion has been proposed as a functional driver of symphyseal fusion diversity. The closely related species *Pr. rubicunda* and *T. cristatus* (formerly *Pr. cristata*) differ in this regard, with fusion of the symphysis observed in the former, a frequent leaper, and but not in the latter (Tague 1993; Washburn 1942). This has been potentially attributed to the need to avoid dislocation of the pubis when landing in *Pr. rubicunda*; the fused pubic symphysis in quadrupedal (e.g., macaques) and brachiating (e.g., gibbons) species, however, make this explanation unlikely (Tague 1993).

Variation in primate pubic symphysis fusion, and particularly the sexual dimorphism in frequency of fusion, may also support an obstetric interpretation. This is reinforced by evidence of sexual dimorphism in the width of the pubic gap in some primate species: a relatively wide gap in females and a tighter joint in males was observed in both *Nycticebus pygmaeus* and *Galago senegalensis* (Torres‐Tamayo et al. 2023). Sexual differences in the frequency and magnitude of pubic openness have been explained as a direct result of higher levels of estrogen in females, particularly in pregnancy, which drive bone resorption and remodeling of the medial margin of the pubis (Rawlins 1975; Tague 1988, 1990). This is supported by the even more extreme pubic remodeling seen in other mammals, such as guinea pigs and pocket gophers, where the pubic symphysis develops into a joint in both males and females, but hormonal changes during sexual maturation or the first pregnancy lead to a dramatic resorption of symphyseal surface in females and a transformation of the fibrous symphyseal cartilage into a more elastic ligament, creating a wide and flexible gap between the pubic bones (Hisaw 1925; Ruth 1936). A similar process occurs in some bats, where females show a pelvis that is widely open at the front, in contrast to the closed symphyseal joint observed in males (Grunstra et al. 2019 and references therein). These examples of sexual dimorphism in the symphyseal form have been interpreted as obstetric adaptations in species that give birth to particularly large neonates; for example, in some bats, the newborn is about 20%–40% the size of the mother (Grunstra et al. 2019).

The lack of fusion in some primate species may be due to the relatively larger brains of primates compared to most mammals, which result in relatively larger neonatal heads compared to birth canal size (Grunstra et al. 2019). This creates a tight cephalopelvic fit (Leutenegger 1974; Schultz 1949), especially for humans. Within this context, the unfused human pubic symphysis has been interpreted as a reversal from the symphyseal fusion seen in other anthropoids and eutherian mammals (Todd 1921a), although this conclusion was based on a comparison across species with very small number of individuals (as low as one per species), sometimes of unknown sex, and often not fully adult. This account fails to explain the presence of pubic fusion in some primate taxa.

Macaques also have high rates of cephalopelvic disproportion (Lovejoy et al. 1997; Morimoto et al. 2023; Tague 1990). The positive relationship between age, parity, and the extent of pubic bone resorption near the pubic symphysis in rhesus monkeys (*Ma. mulatta*) suggests a direct effect of hormonal changes during pregnancy on bone remodeling in this area (Tague 1990). The proposed ultimate, evolutionary explanation for this estrogen‐induced resorption of the pubic bones in female macaques would be inhibition of pubic synostosis, allowing for increased pelvic joint mobility during parturition.

Symphyseal fusion eventually occurs in most macaques, however, and this was originally interpreted as a senile feature (Tasumi 1969). Pubic symphysis fusion in *Ma. fuscata* progresses with age, in a cranial to caudal direction, but less so in females compared to males (Morimoto et al. 2023). Although this fusion leads to a more rigid and obstetrically less favorable pelvis, pelvic remodeling in females during adulthood, which involves the continuing growth of the superior pubic ramus and an anteroposterior expansion of the birth canal, might compensate for the loss of flexibility with age due to symphyseal fusion (Morimoto et al. 2023).

Despite these intriguing evolutionary explanations linking the development of the pubic symphysis with locomotor or obstetric selective pressures in primates, the pattern that has emerged is difficult to interpret. The individuals sampled previously have been, almost exclusively, wild specimens of unknown age, which is problematic when investigating skeletal development. The pubis in humans continues to develop well into the third decade, after the rest of the skeleton has reached full maturation (Dudzik and Langley 2015); as such, it is possible that many of the specimens included in earlier studies had not completed their pelvic development, especially as wild‐caught specimens are (by definition) collected before natural death. Indeed, the frequent pubic symphysis fusion in *Pr. rubicunda* (relative to *Trachypithecus*) has been attributed to the larger proportion of older individuals in the *P. rubicunda* sample (Washburn 1942). The absence of fusion reported for some species in the past might therefore be related to young age and skeletal immaturity, and not be representative of the full range of adult anatomy of the species.

To better evaluate variation in the development of the primate pubic symphysis and the role of obstetric‐related selection, a “wide but shallow” sample of primate pelves was sampled to determine the overall occurrence of fusion across the order. A subsample of specimens of known sex and age available from four different primate species were further examined to determine:

1. the effects of aging on the fusion of the pubic symphysis;
2. the variation in symphyseal fusion in primates, once age is taken into account;
3. the presence of sexual differences in the fusion of the pubic symphysis, as a potential indicator of obstetric adaptation;
4. the link between pregnancy and symphyseal fusion, as a potential proximate explanation of sexual differences in the fusion of the pubic symphysis.

## 2 Materials and Methods

The state of fusion was determined for a large number (*n* = 718) of individuals from 67 nonhuman primate species, to which humans were added as a well‐known species not requiring further data collection (Table S1). Whole‐body computed tomography (CT) scans (obtained after death or from living individuals scanned for other purposes) or osteological material evaluated from visual inspection or CT scans were used throughout. Individuals for which it was not possible to establish whether the pubic symphysis was fused due to the confounding effect of overlaying soft tissue and/or low CT scanning resolution were excluded (Table S1). Due to the nature of the data, we could not distinguish between different types of unfused symphysis, that is, a cartilaginous joint versus a syndesmotic/ligamentous pubic joint; both were described as an unfused or open symphysis.

To evaluate the possible effects of age on fusion, a subsample of specimens with known ages and sex representing four species ( *Galago moholi* , *Macaca mulatta* , *Microcebus murinus* , and *Pan troglodytes* ) (Table 2) was analyzed (captive animals only, as age information is not available for wild individuals). More details of the specimens can be found in the Supporting Information (Table S1).

**TABLE 2:** Number of individuals and life history information for the species included in the subsample for sex‐ and age‐specific analyses of pubic fusion.

Species	F	M	Female first reproduction (years)	Female reproductive senescence (years)	References
*Galago moholi*	7	7	0.4	6.8	Blanco and Zehr 2015
*Macaca mulatta*	72	46	4	22.5	Gagliardi et al. 2007; Pittet, Johnson, and Hinde 2017
*Microcebus murinus*	7	7	0.6	9.9	Blanco and Zehr 2015
*Pan troglodytes*	20	9	10	40	Atzalis and Videan 2009; Videan et al. 2008

To compare the development of the pubic symphysis across species with very different lifespans, age groups standardized by developmental stages were used instead of calendar age for some of the analyses. The age of first female reproduction and the age of female reproductive senescence (Table 2) were used to help define comparable life periods: the reproductive period of life divided into four stages of equal length (stages 1–4), and a postreproductive period of variable length following cessation of reproduction (stage 5, see Table 3). These key life history stages are usually estimated based on observations in captive animals and show some variation across individuals and communities. As such, they can only be considered indicative.

**TABLE 3:** Subdivision in age stages (in years) for the species included in the subsample for sex‐ and age‐specific analyses of pubic fusion.

Species	Stage 1—early reproductive	Stage 2	Stage 3	Stage 4	Stage 5—postreproductive
*Galago moholi*	0.4–2	2.1–3.6	3.7–5.2	5.3–6.8	> 6.8
*Macaca mulatta*	4–8.6	8.7–13.3	13.4–17.9	18–22.5	> 22.5
*Microcebus murinus*	0.6–2.9	3–5.3	5.4–7.6	7.7–9.9	> 9.9
*Pan troglodytes*	10–17.5	17.6–25	25.1–32.5	32.6–40	> 40

**FIGURE 1:**
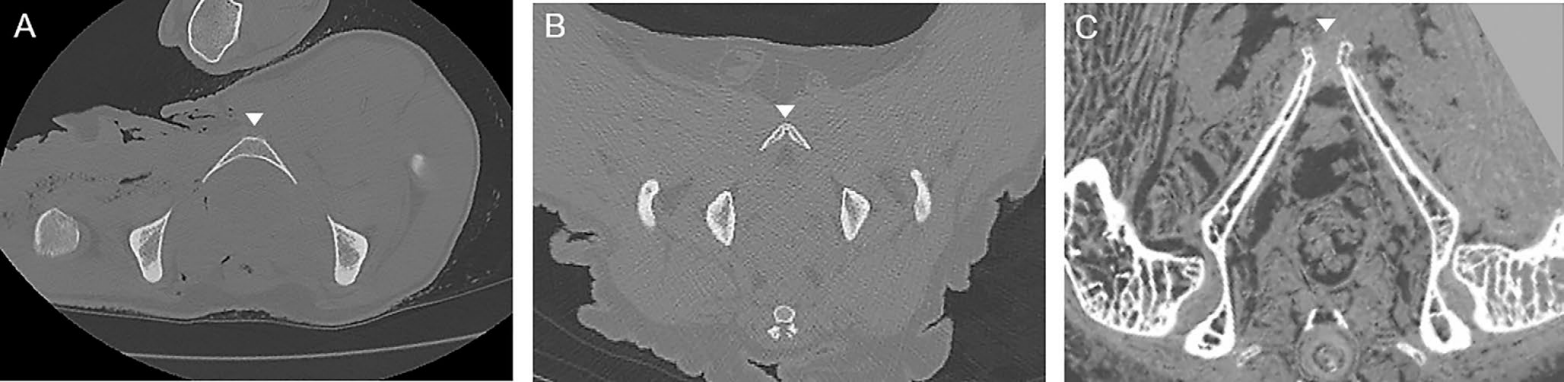
Examination of pubic symphysis condition on CT scan slices, representing a transverse section of the body lying in supine position. The pubic symphysis is indicated by a small triangle. (A) Fully fused pubic bones showing bone remodeling in male *Pa. troglodytes* PRICT‐660; (B) pubic bones showing early stage of fusion in male *Ma. mulatta* UCD‐33734; (C) pubic bones separated by a cartilaginous symphysis in female *Ga. moholi* dlc‐2016f.

**FIGURE 2:**
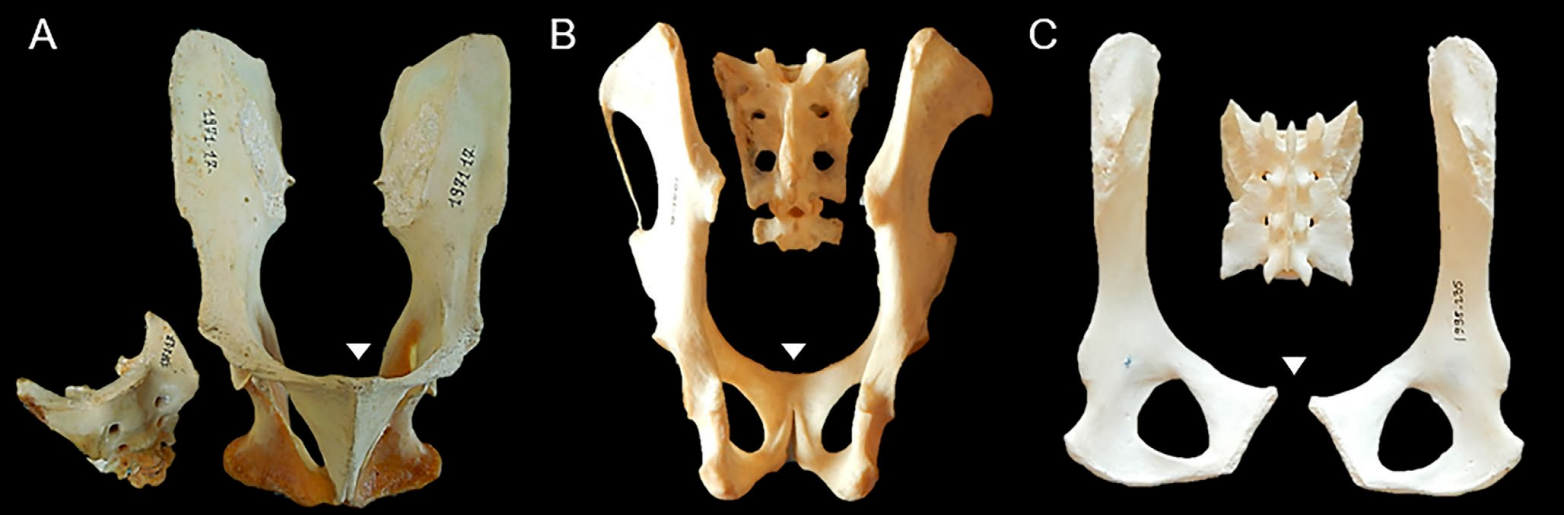
Examples of different levels of fusion of the pubic symphysis in pelvic skeletal material, with the pubic symphysis indicated by a small triangle. (A) Ventral view of two ossa coxae showing a fused pubic symphysis and disarticulated sacrum of male *Mandrillus leucophaeus* MNHN‐ZM‐MO‐1971‐17; (B) Dorsal view of two ossa coxae with a partially fused pubic symphysis and disarticulated sacrum of a female *Propithecus verreauxi* MNHN‐ZM‐MO‐1951‐6; (C) Dorsal view of three pelvic bones with unfused symphysis of female *Alouatta seniculus* MNHN‐ZM‐MO‐1998‐235. Not to scale.

CT scan data of living or cadaveric individuals were examined to determine whether the symphysis was fused or in the process of fusing (Figure 1). The symphysis was categorized as fused if the fusion had occurred at any level of the pubic bodies. For *Ma. mulatta*, for which a larger number of individuals was available across a wide range of ages and more detailed analyses were possible, partial fusion (identified when the fusion had occurred at some level of the pubic bodies, but had not yet led to substantial bone remodeling at the symphysis) was distinguished from the fully fused state to allow for a more in‐depth analysis of the process of fusion. For some individuals of *Pa. troglodytes* and *Ga. moholi*, only skeletal material was available. In these cases, the condition of the symphysis was evaluated from visual inspection (Figure 2) or from CT scans of the bones when evaluation in situ was not possible. Photographs of the pelvis or short videos of CT scan slices for the individuals of these four species are available on the Open Science Framework repository (https://osf.io/tk9mp/).

To evaluate the pattern of changes in the pubic symphysis with age and sex in the four species, the percentage of female and male individuals with a fused symphysis in each age stage was calculated. This simple analysis allowed us to check whether sexual dimorphism is present in the fusion of the symphysis and how the state of the pubic joint changes with age, and to compare the results across our primate species to assess intraspecific and interspecific variation.

The sample size for *Ma. mulatta* was large enough to allow some more in‐depth analyses, which were carried out in R version 4.4.1 (R Core Team 2024). Logistic regression (function “glm”) was used to build models of how the fusion of the pubic symphysis progresses with age, to test whether there is sexual dimorphism in its development and whether the pattern of female development is directly related to the number of conceptions or births experienced during life. Marginal effects (function “margin,” package{margins}; Leeper 2024) were calculated for the variables of interest. The analyses were run using a binary fused/unfused categorization for the status of the pubic symphysis in each individual, and then repeated with more detailed developmental stages (unfused, partial fusion, complete fusion).

## 3 Results

### 3.1 Pubic Symphysis Fusion Across Primates

**FIGURE 3:**
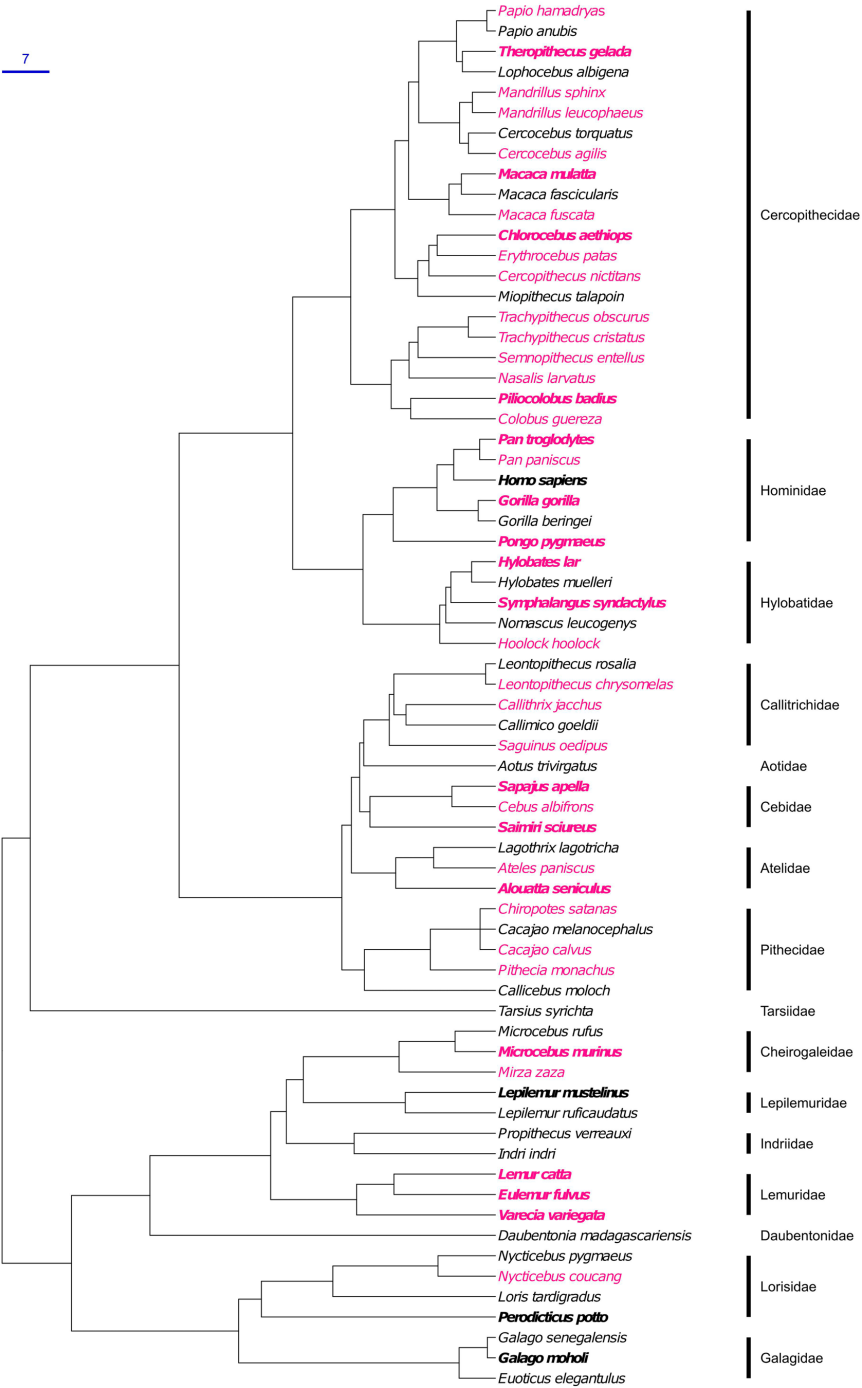
Phylogenetic tree (chronogram) of primate species, color‐coded by symphyseal fusion (pink = observed in at least one individual; black = not observed). Species with at least 10 individuals are in bold. Given the small sample sizes, absence of evidence of symphyseal fusion in most species (except humans) cannot be taken as evidence of the absence of fusion in those taxa. Scale = 7 MY.

Across primates, there is evidence of symphyseal fusion in the large majority (40 out of 68) of species examined (Figure 3, Table 4). Considering the small sample size for most species, these results are only indicative: when fusion is recorded, it indicates that pubic synostosis happens in these species; on the other hand, the absence of evidence of fusion cannot be considered evidence of absence. It is quite possible that the small samples examined for some species included only relatively young adults, and that fusion occurs later in life but was not visible in the available individuals. This is particularly likely for wild specimens, which tend to be caught at a younger age than specimens dying in captivity. The lack of information about age at death makes the pattern of pubic fusion occurrence (or lack thereof) difficult to interpret, and any estimation of frequency of fusion in this wider range of species effectively meaningless. As such, we only report occurrence of fusion as a binary yes/no variable. Even given these caveats, fusion was observed in most primate species across the order, and both in wild and captive specimens.

**TABLE 4:** Occurrence of symphyseal fusion (Y/N) in at least one individual of the studied species, reported by captivity status (captive, wild‐caught, status unknown), with sample size in brackets (*n*). Species with at least one observation of pubic fusion are in bold. Given the small sample sizes for most species, absence of evidence of symphyseal fusion should not be interpreted as evidence of absence in the species. “na”: no specimens available.

Species	Captive (*n*)	Wild (*n*)	Unknown (*n*)	Sample size
***Alouatta seniculus***	N (3)	**Y (5)**	N (2)	10
*Aotus trivirgatus*	N (3)	N (3)	N (1)	7
***Ateles paniscus***	N (3)	**Y (2)**	N (3)	8
***Cacajao calvus***	N (1)	**Y (7)**	na	8
*Cacajao melanocephalus*	na	N (4)	na	4
*Callicebus moloch*	na	N (6)	na	6
*Callimico goeldii*	N (6)	na	N (2)	8
***Callithrix jacchus***	**Y (6)**	na	na	6
***Cebus albifrons***	na	na	**Y (2)**	2
***Cercocebus agilis***	**Y (1)**	na	na	1
*Cercocebus torquatus*	N (1)	N (5)	na	6
***Cercopithecus nictitans***	na	**Y (9)**	na	9
***Chiropotes satanas***	na	na	**Y (1)**	1
***Chlorocebus aethiops***	**Y (6)**	N (2)	**Y (6)**	14
***Colobus guereza***	N (1)	**Y (7)**	na	8
*Daubentonia madagascariensis*	na	N (1)	N (6)	7
***Erythrocebus patas***	**Y (3)**	**Y (3)**	**Y (1)**	7
***Eulemur fulvus***	**Y (20)**	**Y (3)**	**Y (6)**	29
*Euoticus elegantulus*	N (1)	N (6)	na	7
*Galago moholi*	N (14)	na	na	14
*Galago senegalensis*	N (5)	N (2)	N (2)	9
*Gorilla beringei*	na	N (5)	N (1)	6
***Gorilla gorilla***	**Y (2)**	**Y (17)**	**Y (19)**	38
*Homo sapiens*	—	—	N (> 1000)	> 1000
***Hoolock hoolock***	na	na	**Y (2)**	2
***Hylobates lar***	**Y (10)**	**Y (29)**	**Y (2)**	41
*Hylobates muelleri*	na	na	N (1)	1
*Indri indri*	na	N (5)	N (1)	6
*Lagothrix lagotricha*	N (3)	N (2)	N (1)	6
***Lemur catta***	**Y (10)**	na	na	10
***Leontopithecus chrysomelas***	**Y (6)**	na	na	6
*Leontopithecus rosalia*	na	na	N (3)	3
*Lepilemur mustelinus*	na	N (10)	na	10
*Lepilemur ruficaudatus*	na	N (2)	N (3)	5
*Lophocebus albigena*	N (1)	N (2)	N (2)	5
*Loris tardigradus*	na	na	N (1)	1
*Macaca fascicularis*	N (4)	N (1)	N (1)	6
***Macaca fuscata***	**Y (8)**	na	na	8
***Macaca mulatta***	**Y (118)**	na	na	118
***Mandrillus leucophaeus***	**Y (3)**	N (1)	**Y (4)**	8
***Mandrillus sphinx***	**Y (3)**	N (3)	N (1)	7
***Microcebus murinus***	**Y (12)**	**Y (7)**	N (1)	20
*Microcebus rufus*	na	N (3)	na	3
*Miopithecus talapoin*	na	N (1)	N (4)	5
***Mirza zaza***	**Y (8)**	na	na	8
***Nasalis larvatus***	na	**Y (5)**	N (1)	6
*Nomascus leucogenys*	na	na	N (2)	2
***Nycticebus coucang***	**Y (4)**	N (3)	N (2)	9
*Nycticebus pygmaeus*	na	na	N (2)	2
***Pan paniscus***	na	**Y (6)**	na	6
***Pan troglodytes***	**Y (35)**	**Y (12)**	N (4)	51
*Papio anubis*	na	N (6)	N (1)	7
***Papio hamadryas***	N (2)	N (3)	**Y (1)**	6
*Perodicticus potto*	N (3)	N (5)	N (3)	11
***Piliocolobus badius***	na	N (9)	**Y (2)**	11
***Pithecia monachus***	N (1)	**Y (6)**	na	7
***Pongo pygmaeus***	**Y (5)**	N (5)	**Y (3)**	13
*Propithecus verreauxi*	na	N (3)	N (3)	6
***Saguinus oedipus***	**Y (2)**	**Y (3)**	N (2)	7
***Saimiri sciureus***	**Y (3)**	**Y (4)**	N (4)	11
***Sapajus apella***	**Y (4)**	**Y (4)**	N (1)	9
***Semnopithecus entellus***	N (1)	**Y (3)**	**Y (2)**	6
***Symphalangus syndactylus***	**Y (6)**	**Y (4)**	**Y (3)**	13
*Tarsius syrichta*	na	na	N (1)	1
***Theropithecus gelada***	**Y (7)**	na	N (3)	10
***Trachypithecus cristatus***	N (3)	**Y (5)**	na	8
***Trachypithecus obscura***	**Y (1)**	**Y (6)**	N (1)	8
***Varecia variegata***	**Y (7)**	**Y (3)**	**Y (4)**	14

### 3.2 Pubic Symphysis Fusion in Relation to Age and Sex

**FIGURE 4:**
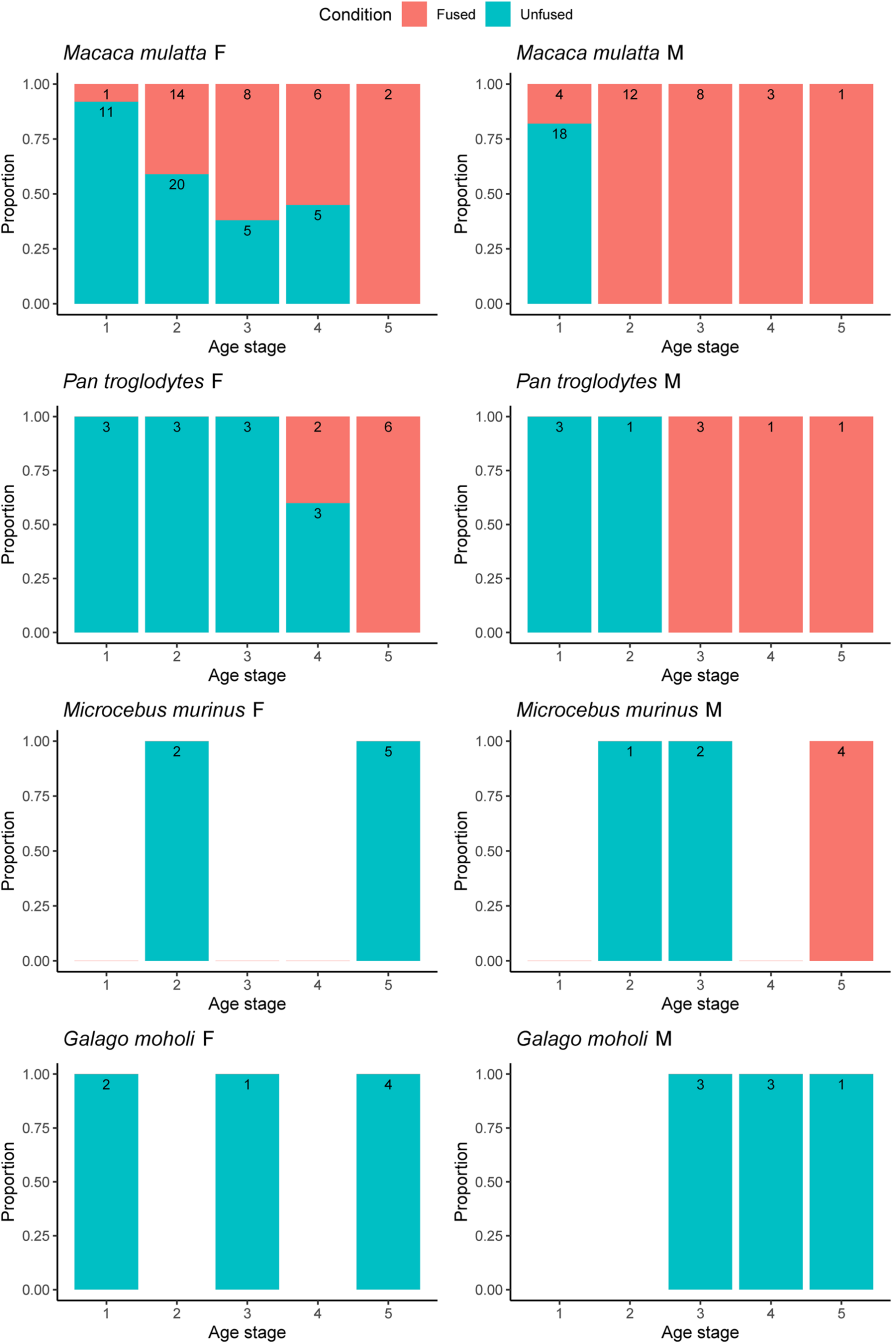
Proportion of fused (red) and unfused (blue) symphyses in the four species by life stage. Numbers in the bars represent the sample size.

When the proportion of individuals with fused and unfused symphyses in each life stage is compared across sexes and across species, it is apparent that, in most species examined in detail here (*Ma. mulatta*, *Pa. troglodytes*, *Mi. murinus*), the symphysis tends to fuse later in life among females than in males (Figure 4). Beyond this shared pattern, however, species differ in the stage at which fusion usually occurs. In chimpanzees, symphyseal fusion in females occurs toward the end of their reproductive life, while in males it happens earlier in adulthood. In macaques, symphyseal fusion starts soon after reaching sexual maturity in both sexes, but while it is complete in all the studied males by stage 2, it only becomes common in females after the middle of their reproductive life (stages 3 and 4), when just over half of females show evidence of fusion. In *Mi. murinus*, fusion occurs in males predominately in the last life stage (although the pattern is less clear due to small sample sizes), while no fusion was observed in females. In *Ga. moholi*, on the other hand, no fusion was observed in either males or females, despite the inclusion of several individuals of postreproductive age, suggesting that, similarly to humans, they preserve an open symphysis throughout life.

**FIGURE 5:**
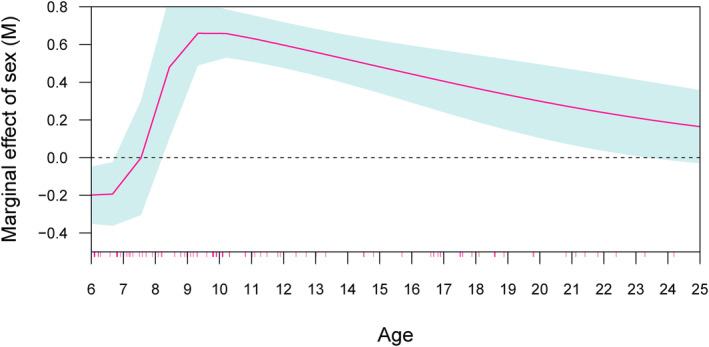
Marginal effects of age by sex (if male) on the probability of symphyseal fusion in *Ma. mulatta*. The 95% confidence interval is represented by the gray shading. The red ticks on the *x* axis indicate the ages of the individuals used in the analysis.

The larger sample of *Ma. mulatta* allows a more detailed investigation of the relationships between sex, age and the state of the symphysis (Table 5). A logistic regression model of the probability of fusion by age and sex and their interaction, with fusion coded in binary fashion (Y = fused, *N* = unfused), indicates that all factors are significant predictors (*p* < 0.05, Supporting Information). The marginal effect of sex by age is visualized in Figure 5. This model breaks down the difference in probability of pubic fusion by sex (if male instead of female) for each year of age (marginal effects at representative cases—also known as MERs). Before age 7 years, both sexes have unfused symphyses, but fusion begins soon after in males. The difference in the probability of fusion between sexes increases rapidly from age seven to a peak around age 10, when males have a probability of fusion that is 0.66 higher than females (SE = 0.07, Supporting Information). Males are significantly more likely to have a fused symphysis between ages 8 and 23, by which time fusion is observed in most individuals of both sexes.

**TABLE 5:** Overall sample size (*N*), age in years and number of individuals by pubic fusion status (*n*) in *Macaca mulatta* .

Sex	*N*	Age (median)	Age (IQR)	Age (min–max)	Unfused (*n*)	Partial fusion (*n*)	Full fusion (*n*)
Female	72	10.9	9.1–17.1	6.1–25.3	41	25	6
Male	46	9.5	7.1–14.5	4–23.3	18	9	19

Abbreviation: IQR, interquartile range.

To test whether sex differences could be explained by the hormonal changes affecting females during pregnancy, the analysis was repeated using the number of conceptions as an additional predictor of pubic fusion. Conception number was not a significant predictor (*p* = 0.071), but the larger model was associated with a lower AIC (Akaike Information Criterion; 109.42 compared with 111.05) than the model without the numbers of conceptions. Age, sex, and their interaction remained significant, suggesting that pregnancies do not explain the sex differences in fusion of the pubic symphysis, although they may contribute to reducing the probability of fusion in females of various ages.

**FIGURE 6:**
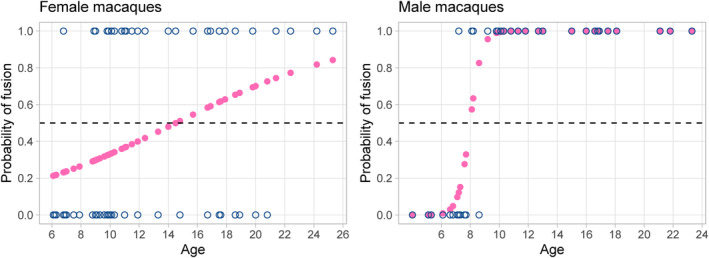
Probability of fusion by age in female (left) and male (right) *Ma. mulatta* as predicted by the logistic model. Observed states of the symphysis in blue open circles.

When analyzing pubic fusion in female and male macaques independently, the difference in the pattern of fusion by age becomes even clearer. Age is a significant predictor of pubic fusion in both sexes, but while in females the probability of fusion per age only reaches 50% after 14.5 years (stage 3, beyond the middle of reproductive life), males reach the same milestone by age eight (stage 2) and with a much steeper increase of probability in early adulthood (Figure 6, Supporting Information).

**FIGURE 7:**
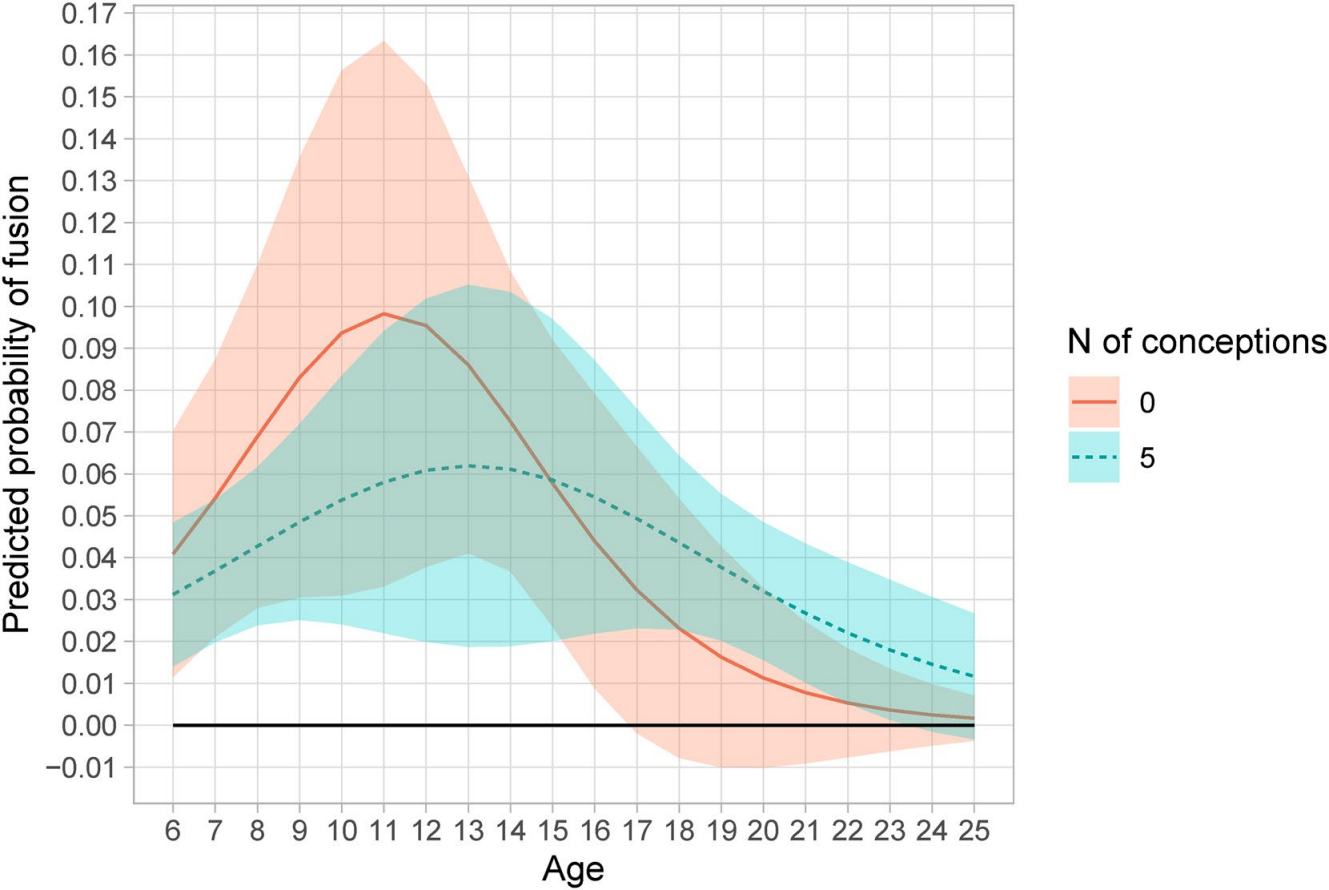
Marginal effects of age at representative numbers of conceptions on pubic fusion of female macaques (*Ma. mulatta*).

Examining the marginal effects of age at representative numbers of conceptions (Figure 7) shows that fusion tends to occur later in life in females with more pregnancies. With zero conceptions, there is a rapid increase in probability of fusion up to age 11, after which the probability remains positive but increases more slowly with additional years of age. In macaques with no conceptions, by age 17, there is no further increase. With five conceptions, the age with the highest increase in probability of fusion is between 12 and 15, declining to a nonsignificant increase by age 23. The effect of larger number of pregnancies (e.g., 10 or more) could not be tested effectively, as very few individuals in that category were present and none in the first two age stages. Based on this analysis, it appears that pregnancy slows down the process of symphyseal fusion.

**FIGURE 8:**
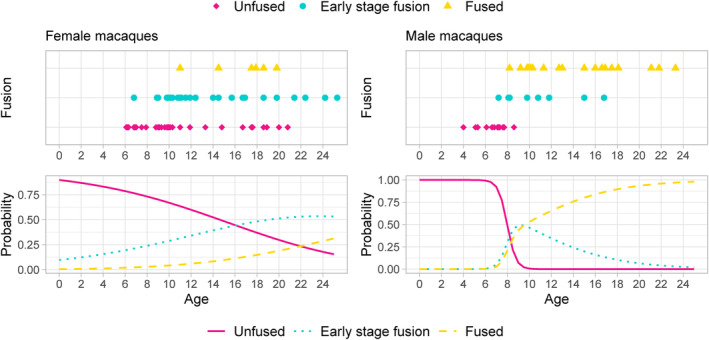
Observed (top) and predicted (bottom) probability of fusion for female (left) and male (right) macaques.

The relatively large number of macaques makes it possible to examine the pattern of symphyseal fusion by age in more detail, by separating the (presumed) earlier stage of fusion (partial fusion, usually occurring more cranially) from full fusion. Figure 8 shows the observed range of fusion by age and the probability of each stage derived by using a multinomial model for females and males (see the Supporting Information for the full results). In females, there is a decrease in the probability of an unfused symphysis with age and a gradual increase in the probability of partial fusion, with full fusion lagging behind and remaining a less likely state even in the oldest individuals. In males, partial fusion appears to be restricted to a much shorter period of life, mostly occurring between seven and 10 years of age. By age 10, most males are expected to have a fully fused symphysis, rising to virtually all males by age 23. These results reiterate the existence of sexual dimorphism in symphyseal development, with a much faster progression to full fusion in males.

## 4 Discussion

### 4.1 Pubic Symphysis Fusion Across Primates

Pubic fusion occurs in most primate species (at least 40 out of 68 species examined) and across all main branches of the order (see Figure 3). It was observed in both captive and wild specimens, indicating that it is a normal outcome of skeletal development and aging in many species and not a pathological consequence of captivity (e.g., due to bone proliferation related to systemic inflammation or metabolic disease). Because of the effects of age, modulated by sex, on symphyseal fusion, a simple measure of frequency of fusion within species is not as informative as one separated by sex and age. For example, a sample with a fairly high proportion of older *Ma. mulatta* individuals in the present study returned a much higher percentage of individuals with fused symphysis (43% of females and 61% of males) than previously reported (1% of females and 23% of males; Lovejoy et al. 1997). As variation in age patterns between these samples is probably driving this difference, comparing species—or sexes within species—using a simple percentage can be misleading.

While pubic symphysis fusion is widespread phylogenetically across the order, the most evident exception seems to be the superfamily Lorisoidea. Pubic fusion was recorded in fewer species in Strepsirhini than Haplorhini overall, but it occurs in several species of lemurs. With the caveat that the absence of evidence is not evidence of absence, an open symphysis might in fact be widespread in lorisoids (Lorisidae and Galagidae, Figure 3). Fusion is absent in *Ga. moholi*, even in old, postreproductive age individuals; the only observation of symphyseal fusion in the family comes from one *N. coucang* female (DPC‐OST‐40), but it is difficult to determine from the micro‐CT scan whether there is symphysis synostosis, or the connective tissue is pathologically ossified (Video S1). Observation of the symphysis in a larger sample of lorisoids that includes individuals of known ages is needed to confirm an open symphysis as a characteristic of this superfamily.

The condition of the pubic symphysis in platyrrhine monkeys is particularly germane, following reports of birth difficulties and the need for external obstetric intervention in some species (Ferraz et al. 2014; Prestes et al. 2014; Varela, Guilló, and Buxó 1995). For instance, in a squirrel monkey (*Sai*. *sciureus*) colony, 16% of offspring were stillborn and 34% perished within the first 100 days of life due to birth‐related injuries (Stoller 1995). These difficulties could be due to this species' high cephalopelvic proportion (Schultz 1949), which would suggest that an unfused symphysis would be advantageous. For example, no fusion had been noted in past studies in *A. azarae* and *Sag*. *geoffroyi*, other platyrrhine species that give birth to relatively large neonates (Tague 2016), as well as in *C. jacchus* (Casteleyn et al. 2012). The present study, however, reveals that pubic fusion occurs in most Platyrrhine monkey species, including *Sai*. *sciureus*, *Sag*. *oedipus*, and *C. jacchus* , reinforcing the conclusion that it is hard to exclude the occurrence of fusion in a species without access to enough individuals of known older ages. These results also show that pubic fusion occurs in species that experience cephalopelvic disproportion and birth difficulties, in contrast to expectations. Almost all Catarrhine monkey species also show fusion of the pubic symphysis, which matches previous observations (Grunstra 2022; Lovejoy et al. 1997; Morimoto et al. 2023; Tague 1993).

Among hominoids, fusion of the pubic symphysis had been documented previously in one specimen of *Gorilla* and one of *Pan*, but none in *Pongo* (Todd 1921b); later work reported no fusion in 49 *Go. gorilla*, although it was seen in *Pa. troglodytes* (Lovejoy et al. 1997). A high prevalence of symphysis fusion has been reported in gibbons (Lovejoy et al. 1997; Tague 2016; Todd 1921b). The present study demonstrates that fusion occurs in effectively all genera of nonhuman hominoids (Figure 3, Table 4, Table S1), regardless of body size, different locomotor modes, or relative neonate size. The exception appears to be *Nomascus*, for which the two individuals examined here were both unfused; given previous evidence of pubic fusion in both sexes of *No. concolor* (Todd 1921b), however, the absence of fusion reported here is likely to be due to our small sample size. In this context, the complete absence of fusion in humans is almost certainly unique within our superfamily. This evolutionary adaptation might reflect changes in pelvic morphology to accommodate the unique demands of bipedal locomotion and childbirth that emerged in our lineage (Grunstra 2023). This is compatible with the lack of fused pubic bones in extinct hominins such as *Australopithecus afarensis* A.L. 288‐1 (Johanson et al. 1982), A*u*. africanus Sts 14 (Robinson 1972), Early/Middle Pleistocene *Homo* KNM‐ER 3228 (Rose 1984) and SH Pelvis 1 (Arsuaga et al. 1999), and Neanderthal specimens Kebara 2 (Rak and Arensburg 1987) and Tabun C1 (McCown and Keith 1939); the sparse fossil record, however, means that any such interpretation should be approached with caution.

Across primates, there appears to be no obvious relationship between evidence of high cephalopelvic proportions and absence of fusion, including in the species analyzed in greater detail here. In particular, high cephalopelvic proportions are found in *Callithrix, Saimiri, Cebus*, and *Macaca* (more or less declining in this order), and *Homo* and *Hylobates* among the apes (Leutenegger 1974; Schultz 1949). The evidence presented here, however, shows pubic fusion in all these species, apart from *Homo*. In contrast, particularly low cephalopelvic proportions have been reported for nonhuman large‐bodied apes (Leutenegger 1974; Schultz 1949); while pubic fusion is present in all these species, it does not appear to be as frequent, or to happen as early in life, as in other anthropoids. It is particularly surprising that *Ma. mulatta* females experience pubic fusion during their reproductive period despite a very tight cephalopelvic fit and reports of difficult birthing (Tinklepaugh and Hartman 1930), while in *Pa. troglodytes* females, fusion is not usually found until the end of their fertile lifespan, despite a lower cephalopelvic ratio.

The contrast between the intraspecific pattern of sexual dimorphism in pubic fusion, implying obstetric adaptation, and the interspecific pattern of no obvious relation between cephalopelvic proportions and pubic fusion, is puzzling. If pubic fusion is an ancestral primate trait, as suggested by its spread across the order, it cannot be interpreted as a new adaptation in the species in which it is observed; in other words, pubic fusion could be informative of obstetric adaptation only in the sense that obstetric‐related selective pressure had not been strong enough to lead to the evolution of a preserved open symphysis during life. It could also be the case that other mechanisms have evolved that facilitate birth in these species, such as the later fusion in females reported here or birth canal expansion through continuous growth of the superior pubic ramus, as identified in macaques (Morimoto et al. 2023). Finally, nonobstetric selective pressures on the pelvis, potentially related to locomotion, might favor a fused pubic symphysis and counteract obstetric pressures for a flexible pelvic girdle. In this sense, what appears to be a lack of a clear interspecific pattern might be the result of different types of obstetrical dilemmas across primate species, balancing in a variety of ways the requirements for the multiple functions of the pelvis. The presence of a preserved open symphysis through life in some primates, on the other hand, can be best interpreted as convergent evolution due to obstetric selective pressure. The only good evidence of this derived trait is currently seen in humans and *Ga. moholi*, but it is quite possible that other species, especially across the superfamily Lorisoidea, show the same adaptation.

### 4.2 Pubic Symphysis Fusion in Relation to Age and Sex

The results of the present study demonstrate that age has a substantial effect on the fusion of the pubic symphysis, with this effect extending well after otherwise complete skeletal development. In some species, fusion occurs early in adulthood (*Ma. mulatta*), in others in middle or late age (*Pa. troglodytes* and *Mi. murinus*), and in others it does not occur at all (*Ga. moholi* and *Ho. sapiens*). A shared pattern of sexual dimorphism in species that display pubic fusion has been identified, with fusion occurring significantly earlier in life in males than in females. This is the opposite of what has been observed for skeletal maturation across the rest of the body, which tends to reach completion earlier in females than males (Dudzik and Langley 2015; Shea 1986; Watts 1975; Zihlman, Bolter, and Boesch 2007). This striking difference in the timing of symphyseal development between the sexes suggests a functional adaptation related to reproduction.

The trend in female chimpanzees of maintaining an unfused pubic symphysis (as a potential obstetric adaptation) until the end of the reproductive stage of life is surprising, considering the lack of cephalopelvic disproportion reported for this species (Schultz 1949; Leutenegger 1974) and, thus, the probable lack of substantial obstetric constraints. Recent re‐examinations of the pelvic morphology of this species, however, point to the existence of sexual dimorphism (Fischer et al. 2021) that might be related to higher cephalopelvic proportions than previously estimated (Laudicina and Cartmill 2023). No median bar (Todd 1921a, 1921b) was detected in any chimpanzees examined in the current study, indicating that the species shows senescent fusion, a consequence of reaching advanced age, as proposed previously (Lovejoy et al. 1997).

The results show that sexual differences in the timing of pubic fusion in macaques cannot be explained solely (or even mainly) by the effect of hormonal changes during pregnancy (*contra* Tague 1990). There is some evidence that elevated estrogen levels during pregnancy might delay pubic synostosis, with fusion more likely to occur later in life in females with multiple conceptions, but the effect seems to be limited and the relationship is not significant. Pubic fusion eventually occurs in most macaques during their reproductive period, and is not a senile feature (*contra* Tasumi 1969). *Ma. mulatta* pubic symphysis fusion progresses with age, but less so in females compared to males (Figure 4), as seen in *Ma. fuscata* (Morimoto et al. 2023), another species which displays cranial to caudal pubic symphysis fusion progress with age, as observed here. Indeed, in some rhesus macaque individuals reported to show “Symphysis fused—early stage” in the present sample (Table S1), only the upper part is fused.

The particularly extreme sexual dimorphism in pubic fusion in *Mi. murinus* is unusual. For this species, there is no evidence of a fused pubic symphysis in any of the females, despite some individuals being well into the postreproductive stage, which supports previous observations of females with an open symphysis (Rasolooarison, Goodman, and Ganzhorn 2000; St Clair 2007). In contrast, males exhibit pubic symphysis fusion in the later stages of life (Figure 4). Strepsirrhines tend to give birth to relatively smaller babies compared to anthropoids (Leutenegger 1973), but *Microcebus* is the smallest extant primate genus and produces proportionally larger offspring (St Clair 2007). This results in a degree of sexual dimorphism in the birth canal that is less commonly observed in larger strepsirhines such as *Arctocebus* and *Perodicticus* (Leutenegger 1973). The fusion of the pubic symphysis in males and its absence in females contributes to overall pelvic sexual dimorphism in this species and is likely an adaptation to mitigate the constraints of giving birth to relatively large babies in this extremely small‐bodied species.

Finally, the present study is the first to report the condition of the pubic symphysis in a lorisoid species, in this case in detail for *Ga. moholi*, revealing a notable lack of fusion in both males and females even in their postreproductive age (Figure 4). This condition is similar to that of humans. There is also sexual dimorphism in the size of the pubic gap, which is considerably wider in female *Ga. moholi* than in males. Sexual dimorphism in the size of the pubic gap has also been observed in *N. pygmaeus* and *Ga. senegalensis*, with a relatively wide gap in females and a tighter joint in males (Torres‐Tamayo et al. 2023). Again, a similar if less pronounced pattern has been observed in humans, with the pubic gap increasing progressively in size when comparing men, nulliparous women, and multiparous women (Alicioglu et al. 2008; Loeschcke 1912; Roberts 1934).

## 5 Conclusion

The development of pubic fusion in adulthood occurs in most primate species, suggesting that it may be the ancestral condition of the order. Fusion tends to happen earlier in life in males than in females in both *Pa. troglodytes* and *Ma. mulatta*, while in *Mi. murinus*, it occurs exclusively in males. This shared pattern of sexual dimorphism suggests an obstetric adaptation, with delayed or no fusion selected for in females through the survival and reproductive advantage of maintaining pelvic flexibility during birth.

Complete lack of pubic fusion in both sexes, even in old age, is not a unique human trait; the same pattern is seen in *Ga. moholi*, and the wider primate data suggest that this could be a common adaptation in Lorisoidea. Unfortunately, small sample sizes and lack of age information do not allow us to confirm lack of fusion in other species with any confidence. It is clear, however, that a preserved open symphysis is a unique human adaptation within Hominoidea. As the only bipedal primate and the most highly encephalized one, both adaptations that required substantial changes in the pelvis, it is only logical to interpret our open symphysis as part of the evolutionary solution to our particular obstetrical dilemma, as a way to alleviate the tight fit of large‐headed neonates in a bipedally adapted, compact pelvis.

## Author Contributions

**Nicole Torres‐Tamayo:** conceptualization (equal), data curation (equal), formal analysis (supporting), funding acquisition (supporting), investigation (equal), methodology (supporting), resources (equal), visualization (equal), writing – original draft (equal), writing – review and editing (equal). **Laura T. Buck:** data curation (equal), funding acquisition (supporting), investigation (equal), methodology (supporting), resources (equal), writing – review and editing (equal). **Eishi Hirasaki:** data curation (equal), funding acquisition (supporting), investigation (equal), methodology (supporting), resources (equal), writing – review and editing (equal). **Todd C. Rae:** conceptualization (equal), data curation (equal), funding acquisition (equal), investigation (equal), methodology (supporting), project administration (supporting), resources (equal), writing – review and editing (equal). **Lia Betti:** conceptualization (equal), data curation (equal), formal analysis (lead), funding acquisition (lead), investigation (equal), methodology (lead), project administration (lead), resources (equal), visualization (equal), writing – original draft (equal), writing – review and editing (equal).

## Supporting information


**Data S1.** Annotated R code used for the analyses.

**Data S2.** Results of logistic regression analyses for Macaca mulatta.

**Table S1.** Details of specimens.

**Video S1.** Video of the CT scan of female *Nycticebus coucang* DPC‐OST‐40, showing potential fusion at the pubic symphysis.

## Data Availability

The data that support the findings of this study are openly available in the Open Science Framework repository at https://osf.io/tk9mp/ and as to the article (screenshots and videos for the four focus species, additional video, data, metadata and R codes). To access the original CT data examined for this study on which the screenshots and videos are based, the authors should contact the institutions where these CT scans were taken. This information is available in Table S1.
