# Supplementary material for: Variation in Pubic Symphysis Fusion Across Primates: Implications for Obstetric Adaptation
Source: Am J Biol Anthropol. 2025 Feb 5;186(2):e25064. doi: 10.1002/ajpa.25064 (PMC11799747; doi:10.1002/ajpa.25064)
Supplement: Supplementary file 1 — Data S1. Annotated R code used for the analyses. [file AJPA-186-e25064-s001.docx]

Annotated code for the analyses ######################################################################à###

### Figure 3: Building a chronogram for primate species - pubic fusion ###

library(ape)

library("caper")

library("geiger")

library(ggtree)

library(ggplot2)

library(cowplot)

library(nnet)

library(VGAM)

library(dplyr)

library(tidyr)

library(ggpubr)

library(margins)

library(prediction)

tree.primates <- read.nexus("consensusTree_10kTrees_Primates_Version3_pubic symphysis.nex") # phylogenetic tree of primates from https://10ktrees.nunn-lab.org/

data.primates <- read.csv("Fusion.table.R.csv") # importing data on fusion of symphysis

Fusion <- comparative.data(phy=tree.primates, data=data.primates, names.col=Binomial) # Combining data on fusion with phylogenetic tree

# set colours of species names based on fusion

fusion.col <- c("black", "deeppink2")[as.factor(Fusion$data$Observed)]

# annotating the tree based on families

plot.phylo(Fusion$phy,cex=0.4, label.offset=0.5, edge.width = 0.5)

nodelabels(cex=0.6, frame = "none") # finding nodes

Families <- data.frame(nodes = c(133,130,18,127,126,125,123,64,115,113,109,2,105,98,94,73),

name.families = c("Galagidae", "Lorisidae","Daubentonidae","Lemuridae","Indriidae","Lepilemuridae","Cheirogaleidae","Tarsiidae","Pithecidae","Cebidae","Callitrichidae","Aotidae","Atelidae","Hominidae","Hylobatidae","Cercopithecidae"))

# Creating the tree annotated with family names

pdf("Fig.3_Tree.pdf", width = 7, height = 10)

ggtree(Fusion$phy, size = 0.2) %<+% data.primates + geom_tiplab(aes(label = Species), size = 2.5, color = fusion.col)+

geom_cladelab(data = Families, mapping = aes(node = nodes, label = name.families),

textcolour = "black" ,barcolour = "black", fontsize = 2.5, align = TRUE, hjust = 'left', offset = 36,offset.text = 1, barsize = 1)+

theme(legend.position = "none")+

xlim(0, 130)+

geom_treescale(x = 0, y = 65, col = "blue3", fontsize = 2.5, linesize = 0.6, offset = 0.4)

dev.off()

##########################################################################################################

### Creation of figure 4: proportion of symphyseal fusion by age group and sex in four focus species ###

data.fusion <- read.csv("Proportions fusion 4 species.csv") # importing table with frequency and proportion of fusion by species, sex and age group

# plot for female P. troglodytes

data.plot <- data.fusion[data.fusion$Species=="Pan troglodytes" & data.fusion$Sex=="F",]

female.chimp <- ggplot(data = data.plot, aes(x = Age.group, y = Percentage, fill = Condition)) +

geom_bar(position="stack", stat="identity")+

labs(x = "Age stage", y = "Proportion") +

theme_classic()+

ggtitle(expression(paste(~ italic("Pan troglodytes"), " F"))) +

geom_text(aes(x = Age.group, y = Percentage, label = ifelse(Number < 1, "", sprintf("%1.0f", Number))), position = "stack", vjust = 1.5, size = 3)+

theme(legend.position = "none")

# plot for male P. troglodytes

data.plot <- data.fusion[data.fusion$Species == "Pan troglodytes" & data.fusion$Sex == "M",]

male.chimp <- ggplot(data = data.plot, aes(x = Age.group, y = Percentage, fill = Condition)) +

geom_bar(position = "stack", stat = "identity")+

labs(x = "Age stage", y = "Proportion") +

theme_classic()+

ggtitle(expression(paste(~ italic("Pan troglodytes"), " M"))) +

geom_text(aes(x = Age.group, y = Percentage, label = ifelse(Number < 1, "", sprintf("%1.0f", Number))), position = "stack", vjust = 1.5, size = 3)+

theme(legend.position = "none")

# plot for female Macaca mulatta

data.plot <- data.fusion[data.fusion$Species == "Macaca mulatta" & data.fusion$Sex == "F",]

female.macaca <- ggplot(data = data.plot, aes(x = Age.group, y = Percentage, fill = Condition)) +

geom_bar(position = "stack", stat = "identity")+

labs(x = "Age stage", y = "Proportion") +

theme_classic()+

ggtitle(expression(paste(~ italic("Macaca mulatta"), " F"))) +

geom_text(aes(x = Age.group, y = Percentage, label = ifelse(Number < 1, "", sprintf("%1.0f", Number))), position = "stack", vjust = 1.5, size = 3)+

theme(legend.position = "none")

# plot for male Macaca mulatta

data.plot <- data.fusion[data.fusion$Species == "Macaca mulatta" & data.fusion$Sex == "M",]

male.macaca <- ggplot(data = data.plot, aes(x = Age.group, y = Percentage, fill = Condition)) +

geom_bar(position = "stack", stat = "identity")+

labs(x = "Age stage", y = "Proportion") +

theme_classic()+

ggtitle(expression(paste(~ italic("Macaca mulatta"), " M"))) +

geom_text(aes(x = Age.group, y = Percentage, label = ifelse(Number < 1, "", sprintf("%1.0f", Number))), position = "stack", vjust = 1.5, size = 3)+

theme(legend.position = "none")

# plot for female Microcebus murinus

data.plot <- data.fusion[data.fusion$Species == "Microcebus murinus" & data.fusion$Sex == "F",]

female.microcebus <- ggplot(data = data.plot, aes(x = Age.group, y = Percentage, fill = Condition)) +

geom_bar(position = "stack", stat = "identity")+

labs(x = "Age stage", y = "Proportion") +

theme_classic()+

ggtitle(expression(paste(~ italic("Microcebus murinus"), " F"))) +

geom_text(aes(x = Age.group, y = Percentage, label = ifelse(Number < 1, "", sprintf("%1.0f", Number))), position = "stack", vjust = 1.5, size = 3)+

theme(legend.position = "none")

# plot for male Microcebus murinus

data.plot <- data.fusion[data.fusion$Species == "Microcebus murinus" & data.fusion$Sex == "M",]

male.microcebus <- ggplot(data = data.plot, aes(x = Age.group, y = Percentage, fill = Condition)) +

geom_bar(position = "stack", stat = "identity")+

labs(x = "Age stage", y = "Proportion") +

theme_classic()+

ggtitle(expression(paste(~ italic("Microcebus murinus"), " M"))) +

geom_text(aes(x = Age.group, y = Percentage, label = ifelse(Number < 1, "", sprintf("%1.0f", Number))), position = "stack", vjust = 1.5, size = 3)+

theme(legend.position = "none")

# plot for female Galago moholi

data.plot <- data.fusion[data.fusion$Species == "Galago moholi" & data.fusion$Sex == "F",]

female.galago <- ggplot(data = data.plot, aes(x = Age.group, y = Percentage, fill = Condition)) +

geom_bar(position = "stack", stat = "identity")+

labs(x = "Age stage", y = "Proportion") +

theme_classic()+

ggtitle(expression(paste(~ italic("Galago moholi"), " F"))) +

geom_text(aes(x = Age.group, y = Percentage, label = ifelse(Number < 1, "", sprintf("%1.0f", Number))), position = "stack", vjust = 1.5, size = 3)+

theme(legend.position="none")

# plot for male Galago moholi

data.plot <- data.fusion[data.fusion$Species=="Galago moholi" & data.fusion$Sex=="M",]

male.galago <- ggplot(data = data.plot, aes(x = Age.group, y = Percentage, fill = Condition)) +

geom_bar(position = "stack", stat = "identity")+

labs(x = "Age stage", y = "Proportion") +

theme_classic()+

ggtitle(expression(paste(~ italic("Galago moholi"), " M"))) +

geom_text(aes(x = Age.group, y = Percentage, label = ifelse(Number < 1, "", sprintf("%1.0f", Number))), position = "stack", vjust = 1.5, size = 3)+

theme(legend.position = "none")

# creating multi-panel figure with all four species

pdf("Fig.4_Proportion.fusion.4.species.pdf", width = 8, height = 12)

ggarrange(female.macaca, male.macaca, female.chimp, male.chimp, female.microcebus, male.microcebus, female.galago, male.galago, ncol = 2, nrow = 4, common.legend = T)

dev.off()

##########################################################################

### Logistic regression analyses in Macaca mulatta and related figures ###

Macaca <- read.csv("Macaca.csv") # Importing data for Macaca mulatta

Macaca <- Macaca[!is.na(Macaca$FusionStageCODE),] #removing NA for fusion symphysis

Macaca$Age<- as.numeric(Macaca$Age)

Macaca$Weight<- as.numeric(Macaca$Weight)

Macaca$Nbirths<- as.integer(Macaca$Nbirths)

Macaca$Nconceptions<- as.integer(Macaca$Nconceptions)

Macaca$Fusion<-as.factor(Macaca$Fusion)

Macaca$Sex<-as.factor(Macaca$Sex)

Macaca$FusionStageCODE<-as.factor(Macaca$FusionStageCODE)

Macaca$FusionStageCODE <- relevel(Macaca$FusionStageCODE , ref = "1")

# A look at the raw data

xtabs(~ Sex + FusionStageCODE, data=Macaca)

xtabs(~ Sex + Fusion, data=Macaca)

## Logistic regression -- All macaques, by Age and Sex

# logistic regression with Y/N fusion by Age and Sex

logistic.Age.Sex.inter <- glm(Fusion ~ Age * Sex, data = Macaca, family = "binomial") # interaction model age and sex

summary(logistic.Age.Sex.inter)

logistic.Age.Sex.Conc.inter <- glm(Fusion ~ Age * Sex + Nconceptions, data = Macaca, family = "binomial") # interaction model plus conceptions

summary(logistic.Age.Sex.Conc.inter)

## Understanding the interaction effects of sex and age

#Average marginal effects (AMEs)

m.Age.Sex <- summary(margins(logistic.Age.Sex.inter)) # only sex

m.Age.Sex

# Marginal effects at representative cases (MERs)

mer.Age.Sex <- summary(margins(logistic.Age.Sex.inter, at = list(Age = 4:25)))

mer.Age.Sex

# Figure 5: Plotting the marginal effect of Sex by age

pdf("Fig.5_Marginal.Sex.Age.Macaques.pdf", width = 8, height = 4)

par(mar = c(4, 4, 1, 1), cex = 1.3)

cplot(logistic.Age.Sex.inter, x = "Age", dx = "SexM", what = "effect",

data = Macaca, xaxt = 'n', cex.axis = 0.8, cex.lab = 1,

ylab="Marginal effect of sex (M)",

col = "deeppink", lwd = 1.5, se.type = "shade", se.fill = "lightcyan2",

xlim = range(6:25), ylim = c(-0.5, 0.8))

axis(1, at = 6:25, cex.axis = 0.8, cex.lab = 1)

abline(h = 0, col = "black", lty = "dashed")

abline(v = 6)

abline(v = 25)

abline(h = 0.8)

dev.off()

# logistic regression with Y/N fusion by Age ONLY IN FEMALES

females <- Macaca[Macaca$Sex=="F",] # subsetting dataset to include only female macaques

logistic.females <- glm(Fusion ~ Age , data = females, family = "binomial") # probability of fusion in femles depending on age

summary(logistic.females)

logistic.females.conc <- glm(Fusion ~ Age + Nconceptions, data = females, family = "binomial") # Age and Number of conceptions

summary(logistic.females.conc)

logistic.females.conc.int <- glm(Fusion ~ Age * Nconceptions, data = females, family = "binomial") # Age and Number of conceptions with interaction effect

summary(logistic.females.conc.int)

# Predicted fusion in females, given the age of the individual.

predicted.data.F <- data.frame(probability.of.fusion = logistic.females$fitted.values, Age = females$Age)

# Figure 6A

F.fusion <- females$Fusion

F.fusion <- ifelse(females$Fusion == "Y", 1, 0)

pdf("Fig.6A_ProbFusionAgeFMacaques.pdf", width = 8, height = 6)

par(mar = c(4, 4, 1, 1), cex = 1.5)

plot(predicted.data.F$probability.of.fusion ~ females$Age,

pch = 21, col = "hotpink", bg = "hotpink",

cex = 0.7, cex.axis = 0.8, cex.lab = 1, xaxt = 'n',

ylab = "Probability of fusion", xlab = "Age", ylim = c(0, 1))

points(F.fusion ~ females$Age, col = "dodgerblue3", cex = 0.7)

axis(1, at = c(6, 8, 10, 12, 14, 16, 18, 20, 22, 24, 26), cex.axis = 0.8)

abline(h = 0.5, col = "black", lty = "dashed")

dev.off()

# Figure 7: Marginal effects at representative cases (MERs) using 0 and 5 conceptions. We excluded higher numbers (e.g. 10 conceptions) as too few individuals were available

mer.F.Age.Conc.No10 <- summary(margins(logistic.females.conc.int, at = list(Nconceptions = c(0, 5), Age = 6:25)))

mer.F.Age.Conc.No10

Age.plot.data <- subset(mer.F.Age.Conc.No10, factor == "Age")

pdf("Fig.7_MERConcept.pdf", width = 6, height = 4)

par(mar = c(4, 4, 1, 4), cex = 2)

ggplot(data = Age.plot.data, aes(x = Age, y = AME, group = Nconceptions)) +

geom_line(aes(colour = factor(Nconceptions), linetype = factor(Nconceptions))) +

scale_colour_manual(values = c("coral2", "cyan4")) +

scale_fill_manual(values=c("coral", "cyan3")) +

geom_ribbon(aes(ymin = lower, ymax = upper, fill = factor(Nconceptions)), linetype = 0, alpha = 0.3) +

labs(x = "Age", y = "Predicted probability of fusion", fill = "N of conceptions", colour = "N of conceptions", linetype = "N of conceptions") +

theme_light() +

scale_x_continuous(breaks = seq(5, 26, 1), minor_breaks = NULL) +

scale_y_continuous(breaks = seq(-0.3, 0.2, 0.01), minor_breaks = NULL) +

geom_line(y = 0)

dev.off()

## Logistic regression with 3 stages of fusion FEMALES only

females$FusionStageCODE <- relevel(females$FusionStageCODE, ref = "2") # sets early fusion as reference

OIM.2 <- multinom(FusionStageCODE ~ 1, data = females) # this is the null model (no predictors)

summary(OIM.2)

logistic.females <- multinom(FusionStageCODE ~ Age , data=females) # actual model includeing age

summary(logistic.females)

z <- summary(logistic.females)$coefficients / summary(logistic.females)$standard.errors # Check the Z-score for the model (wald Z)

z

p <- (1 - pnorm(abs(z), 0, 1)) * 2 # 2-tailed z test

p

anova(OIM.2,logistic.females) # testing whether our model is better than the null model

# Predicted stage of fusion with age in females

predicted.data <- data.frame(probability.of.fusion = logistic.females$fitted.values, Age = females$Age)

# Test the goodness of fit

chisq.test(females$FusionStageCODE,predict(logistic.females))

## Plotting the data (Figure 8 left side)

# Predicting the probabilities

new_data.f <- data.frame(Age = seq(0,25,0.5))

prediction.f <- as.data.frame(predict(logistic.females, new_data.f, type = "probs"))

new_data.f <- cbind(new_data.f, prediction.f[, c(2, 1, 3)])

females$FusionStageCODE <- relevel(females$FusionStageCODE, ref = "1") # sets no fusion as reference

# Plotting the data

par(mar = c(4, 4, 1, 4), cex = 2)

fusion_plot.f <-

ggplot(data = females,aes(x = Age, y = FusionStageCODE)) +

theme_light() +

geom_point(aes(colour = FusionStageCODE, shape = FusionStageCODE), size = 2)+

scale_color_manual(values = c("deeppink2", "cyan3", "gold"), labels = c("Unfused", "Early stage fusion", "Fused")) +

scale_shape_manual(values = c(18, 16, 17), labels = c("Unfused", "Early stage fusion", "Fused")) +

coord_cartesian(xlim = c(0, 25)) +

labs(y = "Fusion", shape = NULL, colour = NULL) +

scale_y_discrete(labels = c(" ", " ", " ")) +

scale_x_continuous(breaks = seq(0, 25, 2), minor_breaks = NULL) +

ggtitle("Female macaques") +

theme(axis.ticks.y.left = element_blank(), legend.position = 'none')

# Plotting the probabilities

par(mar = c(4, 4, 1, 4), cex = 2)

New_data.f <- pivot_longer(data = new_data.f, 2:4, names_to = "FusionStageCODE", values_to = "prob")

prob_plot.f <-

ggplot(data = New_data.f, aes(x = Age, y = prob)) +

scale_colour_manual(values = c("deeppink2", "cyan3", "gold"), labels = c("Unfused", "Early stage fusion", "Fused")) +

scale_linetype_manual(values = c("solid", "dotted", "dashed"), labels = c("Unfused", "Early stage fusion", "Fused")) +

geom_line(linewidth = 0.81, aes(colour = FusionStageCODE, linetype = FusionStageCODE)) +

labs(y = "Probability", linetype = NULL, colour = NULL) +

scale_x_continuous(breaks = seq(0, 25, 2), minor_breaks = NULL) +

theme_light() +

theme(legend.position = 'none')

### logistic regression with Y/N fusion by Age ONLY IN MALES

males <- Macaca[Macaca$Sex == "M", ]

logistic.males <- glm(Fusion ~ Age , data = males, family = "binomial")

summary(logistic.males)

# predicted fusion given the age of the individual.

predicted.data.M <- data.frame(probability.of.fusion = logistic.males$fitted.values, Age = males$Age)

new_data.M <- data.frame(Age = seq(0, 25, 0.5))

prediction.M <- round(predict(logistic.males, new_data.M, type = "response"), 3)

new_data.M <- cbind(new_data.M, prediction.M)

# Figure 6B: Plotting the probability of fusion with age in males

M.fusion <- males$Fusion

M.fusion <- ifelse(males$Fusion == "Y", 1, 0)

pdf("Fig.6B_ProbFusionAgeMMacaques.pdf", width = 8, height = 6)

par(mar = c(4, 4, 1, 1), cex = 1.5)

plot(predicted.data.M$probability.of.fusion~males$Age,

pch = 21, col = "hotpink1", bg = 'hotpink',

cex = 0.7, cex.axis = 0.8, cex.lab = 1, xaxt = 'n',

ylab = "Probability of fusion", xlab = "Age", ylim = c(0, 1))

points(M.fusion ~ males$Age, col = "dodgerblue4", cex = 0.7)

axis(1, at = c(6, 8, 10, 12, 14, 16, 18, 20, 22, 24, 26), cex.axis = 0.8)

abline(h = 0.5, col = "black", lty = "dashed")

dev.off()

# Logistic regression with 3 stages of fusion MALES only

males$FusionStageCODE <- relevel(males$FusionStageCODE, ref = "2") # sets early fusion as reference

OIM.2 <- multinom(FusionStageCODE ~ 1, data = males) # this is the null model (no predictors)

summary(OIM.2)

logistic.males <- multinom(FusionStageCODE ~ Age , data=males) # This is our model of probability of stage of fusion with age

summary(logistic.males)

z <- summary(logistic.males)$coefficients / summary(logistic.males)$standard.errors # Check the Z-score for the model (wald Z)

z

p <- (1 - pnorm(abs(z), 0, 1)) * 2 # 2-tailed z test

p

anova(OIM.2, logistic.males)

## Prediciton based on the age of the individual.

predicted.data.m <- data.frame(probability.of.fusion = logistic.males$fitted.values, Age = males$Age)

# Test the goodness of fit

chisq.test(males$FusionStageCODE, predict(logistic.males))

## Plotting the data (Figure 8 right side)

males$FusionStageCODE <- relevel(males$FusionStageCODE, ref = "1") # sets no fusion as reference

# Predicting the probabilities

new_data.m <- data.frame(Age = seq(0, 25, 0.5))

prediction.m <- as.data.frame(predict(logistic.males, new_data.m, type = "probs"))

new_data.m <- cbind(new_data.m, prediction.m[ , c(2, 1, 3)])

# Plotting the data

par(mar = c(4, 4, 1, 4), cex = 2)

fusion_plot.m <-

ggplot(data = males,aes(x = Age, y = FusionStageCODE)) +

theme_light() +

geom_point(aes(colour = FusionStageCODE,shape = FusionStageCODE),size=2) +

scale_color_manual(values = c("deeppink2", "cyan3", "gold"), labels = c("Unfused", "Early stage fusion", "Fused")) +

scale_shape_manual (values = c(18, 16, 17), labels = c("Unfused", "Early stage fusion", "Fused")) +

coord_cartesian(xlim = c(0, 25)) +

labs(y="Fusion", shape = NULL, colour = NULL) +

scale_y_discrete(labels = c(" ", " ", " ")) +

scale_x_continuous(breaks = seq(0, 25, 2), minor_breaks=NULL) +

ggtitle("Male macaques") +

theme(axis.ticks.y.left = element_blank(), legend.position = 'none')

# Plotting the probabilities

par(mar = c(4, 4, 1, 4), cex = 2)

New_data.m <- pivot_longer(data = new_data.m, 2:4, names_to = "FusionStageCODE", values_to = "prob")

prob_plot.m <-

ggplot(data = New_data.m,aes(x = Age, y = prob)) +

scale_colour_manual(values = c("deeppink2", "cyan3", "gold"), labels = c("Unfused", "Early stage fusion", "Fused")) +

scale_linetype_manual(values = c("solid", "dotted", "dashed"), labels = c("Unfused", "Early stage fusion", "Fused")) +

geom_line(linewidth = 0.81, aes(colour = FusionStageCODE, linetype = FusionStageCODE)) +

labs(y = "Probability", linetype = NULL, colour = NULL) +

scale_x_continuous(breaks = seq(0, 25, 2), minor_breaks = NULL) +

theme_light()+

theme(legend.position = 'none')

# Merging the two plots for males and females (Figure 8)

female.male.3.stages.plot1 <- ggarrange(

ggplot(data = females, aes(x = Age, y = FusionStageCODE)) +

theme_light()+

geom_point(aes(colour = FusionStageCODE,shape = FusionStageCODE), size = 2)+

scale_color_manual(values = c("deeppink2", "cyan3", "gold"), labels = c("Unfused", "Early stage fusion", "Fused"))+

scale_shape_manual (values = c(18, 16, 17), labels = c("Unfused", "Early stage fusion", "Fused"))+

coord_cartesian(xlim = c(0, 25)) +

labs(y = "Fusion", x = NULL, shape = NULL, colour = NULL) +

scale_y_discrete(labels = c(" ", " ", " ")) +

scale_x_continuous(breaks = seq(0, 25, 2), minor_breaks = NULL) +

ggtitle("Female macaques") +

theme(legend.text = element_text(size = 11), axis.ticks.y.left = element_blank(), axis.title.y = element_text(size = 11), plot.title = element_text(size = 11)),

ggplot(data = males, aes(x = Age, y = FusionStageCODE)) +

theme_light()+

geom_point(aes(colour = FusionStageCODE,shape = FusionStageCODE),size=2) +

scale_color_manual(values = c("deeppink2", "cyan3", "gold"), labels = c("Unfused", "Early stage fusion", "Fused")) +

scale_shape_manual values = c(18, 16, 17), labels = c("Unfused", "Early stage fusion", "Fused")) +

coord_cartesian(xlim = c(0, 25)) +

labs(y = "Fusion", x = NULL, shape = NULL, colour = NULL) +

scale_y_discrete(labels = c(" ", " ", " ")) +

scale_x_continuous(breaks = seq(0, 25, 2), minor_breaks = NULL) +

ggtitle("Male macaques") +

theme(legend.text = element_text(size = 11), axis.ticks.y.left = element_blank(), axis.title.y = element_text(size = 11), plot.title = element_text(size = 11)),

common.legend = T,

legend = "top")

female.male.3.stages.plot2 <- ggarrange(

ggplot(data = New_data.f, aes(x = Age, y = prob)) +

scale_colour_manual(values = c("deeppink2", "cyan3", "gold"), labels = c("Unfused", "Early stage fusion", "Fused")) +

scale_linetype_manual(values = c("solid", "dotted", "dashed"), labels = c("Unfused", "Early stage fusion", "Fused")) +

geom_line(linewidth = 0.7, aes(colour = FusionStageCODE, linetype = FusionStageCODE)) +

labs(y = "Probability", linetype = NULL, colour = NULL) +

scale_x_continuous(breaks = seq(0, 25, 2), minor_breaks = NULL) +

theme_light() +

theme(legend.text = element_text(size = 11), axis.title.y = element_text(size = 11), axis.title.x = element_text(size = 11)),

ggplot(data = New_data.m,aes(x = Age, y = prob))+

scale_colour_manual(values = c("deeppink2", "cyan3", "gold"), labels = c("Unfused", "Early stage fusion", "Fused")) +

scale_linetype_manual(values = c("solid", "dotted", "dashed"), labels = c("Unfused", "Early stage fusion", "Fused")) +

geom_line(linewidth = 0.7,aes(colour = FusionStageCODE, linetype = FusionStageCODE)) +

labs(y = "Probability", linetype = NULL, colour = NULL) +

scale_x_continuous(breaks = seq(0, 25, 2), minor_breaks = NULL) +

theme_light() +

theme(legend.text = element_text(size = 11), axis.title.y = element_text(size = 11), axis.title.x = element_text(size = 11)),

common.legend = T,

legend = "bottom")

##Full picture (Figure 8)

pdf("Fig.8_both.sexes.3stages.pdf", width = 8, height = 4)

ggarrange(female.male.3.stages.plot1, female.male.3.stages.plot2, nrow = 2)

dev.off()
