# Supplementary material for: Variation in Pubic Symphysis Fusion Across Primates: Implications for Obstetric Adaptation
Source: Am J Biol Anthropol. 2025 Feb 5;186(2):e25064. doi: 10.1002/ajpa.25064 (PMC11799747; doi:10.1002/ajpa.25064)
Supplement: Supplementary file 2 — Data S2. Results of logistic regression analyses for Macaca mulatta. [file AJPA-186-e25064-s003.docx]

Torres-Tamayo et al. (submitted) Variability in pubic symphysis fusion across Primates: implications for obstetric evolution. Am J Biol Anthropol

**Supporting information - Results logistic regressions for *Macaca mulatta***

**Sample size = 118**

**Pubic fusion in the sample (Yes/No) by sex**

**Sex N Y**

F 41 31

M 18 28

**Pubic fusion in the sample by sex using three stages of pubic fusion:**

1 = unfused

2 = early stage of fusion

3 = fusion complete

**Sex 1 2 3**

F 41 25 6

M 18 9 19

**Logistic regression of pubic fusion (Y/N) depending on Age and Sex and their interaction**

Call:

glm(formula = Fusion ~ Age * Sex, family = "binomial", data = Macaca)

Coefficients:

Estimate Std. Error z value Pr(>|z|)

(Intercept) -2.25697 0.74261 -3.039 0.00237 **

Age 0.15544 0.05494 2.829 0.00467 **

SexM -17.89321 8.05954 -2.220 0.02641 *

Age:SexM 2.36904 1.03098 2.298 0.02157 *

---

Signif. codes: 0 ‘***’ 0.001 ‘**’ 0.01 ‘*’ 0.05 ‘.’ 0.1 ‘ ’ 1

(Dispersion parameter for binomial family taken to be 1)

Null deviance: 163.58 on 117 degrees of freedom

Residual deviance: 103.05 on 114 degrees of freedom

AIC: 111.05

Number of Fisher Scoring iterations: 9

**Marginal effect of sex (male) by age**

factor Age AME SE z p lower upper

SexM 4.0000 -0.1631 0.0739 -2.2066 0.0273 -0.3079 -0.0182

SexM 5.0000 -0.1849 0.0746 -2.4798 0.0131 -0.3311 -0.0388

SexM 6.0000 -0.2034 0.0753 -2.6998 0.0069 -0.3511 -0.0557

SexM 7.0000 -0.1597 0.1045 -1.5279 0.1265 -0.3646 0.0452

SexM 8.0000 0.2451 0.1961 1.2497 0.2114 -0.1393 0.6295

SexM 9.0000 0.6312 0.1173 5.3824 0.0000 0.4013 0.8610

SexM 10.0000 0.6627 0.0668 9.9210 0.0000 0.5318 0.7936

SexM 11.0000 0.6330 0.0629 10.0639 0.0000 0.5097 0.7563

SexM 12.0000 0.5967 0.0619 9.6446 0.0000 0.4754 0.7179

SexM 13.0000 0.5588 0.0628 8.8982 0.0000 0.4357 0.6819

SexM 14.0000 0.5202 0.0659 7.8938 0.0000 0.3910 0.6494

SexM 15.0000 0.4814 0.0709 6.7924 0.0000 0.3425 0.6203

SexM 16.0000 0.4427 0.0770 5.7475 0.0000 0.2918 0.5937

SexM 17.0000 0.4048 0.0836 4.8415 0.0000 0.2409 0.5687

SexM 18.0000 0.3680 0.0899 4.0933 0.0000 0.1918 0.5442

SexM 19.0000 0.3326 0.0953 3.4891 0.0005 0.1458 0.5195

SexM 20.0000 0.2991 0.0995 3.0044 0.0027 0.1040 0.4942

SexM 21.0000 0.2675 0.1023 2.6150 0.0089 0.0670 0.4680

SexM 22.0000 0.2382 0.1036 2.3001 0.0214 0.0352 0.4411

SexM 23.0000 0.2111 0.1033 2.0433 0.0410 0.0086 0.4137

SexM 24.0000 0.1864 0.1017 1.8321 0.0669 -0.0130 0.3858

SexM 25.0000 0.1640 0.0990 1.6566 0.0976 -0.0300 0.3580

**Logistic regression of pubic fusion (Y/N) depending on Age and Sex and their interaction, plus number of conceptions**

Call:

glm(formula = Fusion ~ Age * Sex + Nconceptions, family = "binomial",

data = Macaca)

Coefficients:

Estimate Std. Error z value Pr(>|z|)

(Intercept) -2.80917 0.85303 -3.293 0.000991 ***

Age 0.26173 0.08641 3.029 0.002453 **

SexM -17.34100 8.07047 -2.149 0.031658 *

Nconceptions -0.20144 0.11170 -1.804 0.071310 .

Age:SexM 2.26274 1.03314 2.190 0.028512 *

---

Signif. codes: 0 ‘***’ 0.001 ‘**’ 0.01 ‘*’ 0.05 ‘.’ 0.1 ‘ ’ 1

(Dispersion parameter for binomial family taken to be 1)

Null deviance: 163.583 on 117 degrees of freedom

Residual deviance: 99.416 on 113 degrees of freedom

AIC: 109.42

Number of Fisher Scoring iterations: 9

**RESULTS FOR FEMALE MACAQUES ONLY**

**Probability of fusion (Y/N) in females depending on age**

Call:

glm(formula = Fusion ~ Age, family = "binomial", data = females)

Coefficients:

Estimate Std. Error z value Pr(>|z|)

(Intercept) -2.25697 0.74261 -3.039 0.00237 **

Age 0.15544 0.05494 2.829 0.00467 **

---

Signif. codes: 0 ‘***’ 0.001 ‘**’ 0.01 ‘*’ 0.05 ‘.’ 0.1 ‘ ’ 1

(Dispersion parameter for binomial family taken to be 1)

Null deviance: 98.420 on 71 degrees of freedom

Residual deviance: 89.312 on 70 degrees of freedom

AIC: 93.312

Number of Fisher Scoring iterations: 4

**probability.of.fusion Age**

0.2126880 6.1

0.2153025 6.2

0.2153025 6.2

0.2179402 6.3

0.2314767 6.8

0.2314767 6.8

0.2314767 6.8

0.2342534 6.9

0.2370531 7.0

0.2370531 7.0

0.2513951 7.5

0.2632760 7.9

0.2912922 8.8

0.2912922 8.8

0.2945115 8.9

0.2977514 9.0

0.2977514 9.0

0.2977514 9.0

0.3010117 9.1

0.3075928 9.3

0.3075928 9.3

0.3176124 9.6

0.3243880 9.8

0.3243880 9.8

0.3243880 9.8

0.3243880 9.8

0.3278039 9.9

0.3278039 9.9

0.3312380 10.0

0.3312380 10.0

0.3346903 10.1

0.3346903 10.1

0.3416478 10.3

0.3416478 10.3

0.3416478 10.3

0.3593375 10.8

0.3665251 11.0

0.3665251 11.0

0.3665251 11.0

0.3665251 11.0

0.3701416 11.1

0.3847501 11.5

0.3995695 11.9

0.3995695 11.9

0.4183525 12.4

0.4527300 13.3

0.4798010 14.0

0.4992197 14.5

0.4992197 14.5

0.5108758 14.8

0.5457230 15.7

0.5839092 16.7

0.5839092 16.7

0.5914419 16.9

0.6137712 17.5

0.6137712 17.5

0.6174493 17.6

0.6174493 17.6

0.6284019 17.9

0.6534352 18.6

0.6534352 18.6

0.6534352 18.6

0.6534352 18.6

0.6639183 18.9

0.6943847 19.8

0.6943847 19.8

0.7009418 20.0

0.7263410 20.8

0.7444827 21.4

0.7729137 22.4

0.8182633 24.2

0.8423219 25.3

**Including both Age and Number of conceptions**

Call:

glm(formula = Fusion ~ Age + Nconceptions, family = "binomial",

data = females)

Coefficients:

Estimate Std. Error z value Pr(>|z|)

(Intercept) -2.80917 0.85302 -3.293 0.000991 ***

Age 0.26173 0.08641 3.029 0.002453 **

Nconceptions -0.20144 0.11170 -1.804 0.071310 .

---

Signif. codes: 0 ‘***’ 0.001 ‘**’ 0.01 ‘*’ 0.05 ‘.’ 0.1 ‘ ’ 1

(Dispersion parameter for binomial family taken to be 1)

Null deviance: 98.420 on 71 degrees of freedom

Residual deviance: 85.679 on 69 degrees of freedom

AIC: 91.679

Number of Fisher Scoring iterations: 4

**Including the interaction between age and number of conceptions**

Call:

glm(formula = Fusion ~ Age * Nconceptions, family = "binomial",

data = females)

Coefficients:

Estimate Std. Error z value Pr(>|z|)

(Intercept) -4.37252 1.43883 -3.039 0.00237 **

Age 0.39313 0.13200 2.978 0.00290 **

Nconceptions 0.22675 0.29760 0.762 0.44610

Age:Nconceptions -0.02908 0.01926 -1.510 0.13113

---

Signif. codes: 0 ‘***’ 0.001 ‘**’ 0.01 ‘*’ 0.05 ‘.’ 0.1 ‘ ’ 1

(Dispersion parameter for binomial family taken to be 1)

Null deviance: 98.420 on 71 degrees of freedom

Residual deviance: 83.308 on 68 degrees of freedom

AIC: 91.308

Number of Fisher Scoring iterations: 4

**Probability of fusion (3 stages) in females depending on age. Fusion stage 2 (early fusion) set as reference value**

Call:

multinom(formula = FusionStageCODE ~ Age, data = females)

Coefficients:

(Intercept) Age

1 2.228608 -0.13860197

3 -2.844334 0.09244931

Std. Errors:

(Intercept) Age

1 0.7738943 0.05743723

3 1.5224959 0.09032280

Residual Deviance: 118.6932

AIC: 126.6932

**RESULTS FOR MALE MACAQUES ONLY**

**Probability of fusion (Y/N) in males depending on age**

Call:

glm(formula = Fusion ~ Age, family = "binomial", data = males)

Coefficients:

Estimate Std. Error z value Pr(>|z|)

(Intercept) -20.150 8.025 -2.511 0.0120 *

Age 2.524 1.030 2.452 0.0142 *

---

Signif. codes: 0 ‘***’ 0.001 ‘**’ 0.01 ‘*’ 0.05 ‘.’ 0.1 ‘ ’ 1

(Dispersion parameter for binomial family taken to be 1)

Null deviance: 61.578 on 45 degrees of freedom

Residual deviance: 13.736 on 44 degrees of freedom

AIC: 17.736

Number of Fisher Scoring iterations: 9

Age probability.of.fusion

1 0.0 0.000

2 0.5 0.000

3 1.0 0.000

4 1.5 0.000

5 2.0 0.000

6 2.5 0.000

7 3.0 0.000

8 3.5 0.000

9 4.0 0.000

10 4.5 0.000

11 5.0 0.001

12 5.5 0.002

13 6.0 0.007

14 6.5 0.023

15 7.0 0.077

16 7.5 0.229

17 8.0 0.511

18 8.5 0.787

19 9.0 0.929

20 9.5 0.979

21 10.0 0.994

22 10.5 0.998

23 11.0 1.000

24 11.5 1.000

25 12.0 1.000

26 12.5 1.000

27 13.0 1.000

28 13.5 1.000

29 14.0 1.000

30 14.5 1.000

31 15.0 1.000

32 15.5 1.000

33 16.0 1.000

34 16.5 1.000

35 17.0 1.000

36 17.5 1.000

37 18.0 1.000

38 18.5 1.000

39 19.0 1.000

40 19.5 1.000

41 20.0 1.000

42 20.5 1.000

43 21.0 1.000

44 21.5 1.000

45 22.0 1.000

46 22.5 1.000

47 23.0 1.000

48 23.5 1.000

49 24.0 1.000

50 24.5 1.000

51 25.0 1.000

**Probability of fusion (3 stages) in males depending on age. Fusion stage 2 (early fusion) set as reference value**

multinom(formula = FusionStageCODE ~ Age, data = males)

Coefficients:

(Intercept) Age

1 19.859132 -2.4222251

3 -2.395525 0.2526091

Std. Errors:

(Intercept) Age

1 8.044890 1.0318137

3 1.602989 0.1323997

Residual Deviance: 43.90773

AIC: 51.90773
